# Structural Conformation Comparison of Different Clear Aligner Systems: An In Vitro Study

**DOI:** 10.3390/dj10050073

**Published:** 2022-04-30

**Authors:** Aseel Alhendi, Rita Khounganian, Raisuddin Ali, Saeed Ali Syed, Abdullazez Almudhi

**Affiliations:** 1 Department of Pediatric Dentistry and Orthodontics, College of Dentistry, King Saud University, Riyadh 11545, Saudi Arabia; abalmudhi@ksu.edu.sa; 2 Department of Oral Medicine and Diagnostic Sciences, College of Dentistry, King Saud University, Riyadh 11545, Saudi Arabia; ritak@ksu.edu.sa; 3 Department of Pharmaceutics, College of Pharmacy, King Saud University, Riyadh 11451, Saudi Arabia; ramohammad@ksu.edu.sa; 4 Department of Pharmaceutical Chemistry, College of Pharmacy, King Saud University, Riyadh 11451, Saudi Arabia; sasyed@ksu.edu.sa

**Keywords:** Clear Aligner Appliances, Fourier transform infrared spectroscopy, Vickers hardness, scanning electron microscopy, EDX-microanalysis

## Abstract

The aim of this study was to evaluate the structural conformations of three clear aligner systems, Eon^®^, SureSmile^®^, and Clarity^®^, and compare them with the most commonly used system, Invisalign^®^. Clear aligner samples from Invisalign^®^, Eon^®^, SureSmile^®^, and Clarity^®^ were cut into 5 × 5 mm squares and exposed to artificial saliva for 2 weeks. The specimens were then subjected to a Vickers hardness test by applying three separate indentations with a 25 gf load for 15 s. Hardness was calculated using the following formula: Vickers hardness number = 1.854 (F/D2). Fourier transform infrared spectroscopy (FTIR) analysis was performed, with a diamond hemisphere and infrared beam being allowed to pass through each specimen. A mid-infrared range from 4000 to 375 cm^−1^ was recorded. The samples were also evaluated using scanning electron microscopy (SEM) combined with energy-dispersive X-ray microanalysis spectroscopy at different magnifications. No statistically significant differences were observed between the included systems with regard to hardness. All systems showed a polyurethane-based material, as illustrated by the FTIR analysis. Some structural variations were noted in the Invisalign^®^ system, which had a more homogeneous architecture. Statistically significant differences in the carbon weights were found among the systems. The four systems presented comparable hardness levels. Mild molecular composition differences were found, but all systems had the similarity of being composed of a polyurethane-based material. Carbon and oxygen were the main elements, as they were located in all studied clear aligners. The SEM analysis revealed that Invisalign^®^ had a smoother surface than the other three systems. All included clear aligners had similar characteristics with minimal differences, providing a wide variety of options for clinical orthodontic treatment according to patients’ demands.

## 1. Introduction

Since the late 1990s, invisible tooth correction technology has been established as an alternative to conventional fixed orthodontic treatment. A wide variety of clear aligner systems are commercially available worldwide. Each manufacturing company asserts its own production material to have superior clinical effectiveness with improved characteristics [1,2,3]. Align Technology is considered the leading company in the clear aligner market, producing the world’s most advanced clear aligner system (Invisalign^®^) from SmartTrack material [4]. Meanwhile, Eon^®^ Holdings, established in 2011, designs and manufactures clear removable aligners with a special form of polyurethane [5]. In 2018, 3M^™^ launched their own clear aligner system called Clarity^®^, made from durable and virtually invisible material [6]. Lastly, SureSmile^®^ aligners were designed by Dentsply-Sirona in 2019. They are produced from Essix plastic, which is a thermoformed polyurethane material [7].

Although most invisible aligners are polyurethane thermosetting polymeric products, some variations exist among different companies. These differences are attributed to processing variations in the manufacturing techniques that incorporate various additives and dimensional characteristics [8].

The oral cavity is considered a unique environment because of the presence of oral flora and its byproducts. Hence, clear appliances’ exposure and prolonged contact with the intraoral environment (saliva, enzymes, bacteria, and bacterial byproducts), various temperatures, and pH concentrations as well as trauma caused by speech, swallowing, and bruxism may adversely affect the composition of the material and subsequently result in surface alterations [9,10].

Thavarajah and Thennukonda (2015) reported surface and molecular changes in clear aligner systems. The structural changes of the materials were dependent on the characteristics and composition of the outer monolayer [11]. Modification of the surface and properties of the material can enhance the longevity of the appliance, including aging of the polymeric material, contributing to changes in mechanical properties, such as friction and super-elasticity.

It was previously reported that the absorption of liquids can cause major structural alterations of polyurethane, inducing a plasticizing effect and a subsequent decrease in crack resistance [12]. Previous studies have concluded that exposure to the oral environment leads to morphological, structural, and compositional changes that in turn affect the mechanical properties of dental polymers.

Hardness is a mechanical property that indicates the ability of a material surface to withstand local deformation, and it is measured by applying a certain load for a specific time using a micro-hardness tester. Vickers indenters have been used as a standard method for material characterization because they are inexpensive, simple, and allow for non-destructive analysis [13]. On the basis of previous investigations, the hardness of a polymeric material has been found to be sensitive to residual monomer content in addition to material thickness [14,15]. Hence, the thickness of polymeric materials plays a role in force delivery, and its effect on the deflection amount and structural characteristics may also produce force delivery changes. In 2004, Shuster and his colleagues reported a significant increase in aligner stiffness after intraoral exposure, attributed to chewing forces and salivary enzymes [9].

To the best of our knowledge, the material properties of the Eon^®^, Clarity^®^, and SureSmile^®^ systems have not been previously investigated. This raises the following question: do different clear aligners’ materials have similar characteristics in relation to structural conformation? Therefore, the aim of this study was to compare the structural conformation of three different clear aligner systems (Eon^®^, SureSmile^®^, and Clarity^®^) with the most commonly used system, Invisalign^®^. Our null hypothesis was that there would be no statistically significant differences in the structural conformation among the studied variables of the four systems.

## 2. Materials and Methods

*Characterization of Specimens*: Maxillary and mandibular clear aligners from all four included systems (Invisalign^®^, Eon^®^, SureSmile^®^, and Clarity^®^) were cut into 5 × 5 mm squares. The samples were exposed to freshly prepared artificial saliva with pH = 6.7 and incubated in laboratory glass dishes for 2 weeks at 37 °C to simulate the intraoral aging process.

All specimens were then subjected to the tests described below.

### 2.1. Vickers Indenter

Each specimen was mounted in a plastic mold using an orthodontic plaster to facilitate the indentation process. The microhardness was tested using a Vickers indenter (INNOVATEST, FALCON 450, Deutschland GmbH) by applying a 25 gf load for 15 s. Three indentations were made at the center of each specimen. The indentations were approximately 100 μm apart. The size of each indentation was measured using a microscope. The Vickers hardness number (VHN) was calculated using the following formula:

VHN = 1.854(F/D2)

where F is the applied load measured in kilogram-force and D2 is the area of the indentation measured in square millimeters.

### 2.2. Fourier Transform Infrared Spectroscopy

The Fourier Transform Infrared (FTIR) spectra of the four clear aligner systems were recorded using an Alpha spectrophotometer (Bruker, Germany) equipped with a platinum attenuated total reflection module and a diamond hemisphere. Instrument control and data recording and processing were performed using OPUS version 7.8 (Bruker Optik GmbH, Germany). The flat portion of the small samples was placed on the sample holder touching the diamond hemisphere, and the infrared (IR) beam was allowed to pass through the diamond hemisphere to create at least one reflectance from the surface of contact with the specimen. The data were recorded in the mid-infrared range of 4000 to 375 cm^−^^1^ with a spectral resolution of 2 cm^−^^1^. The software recorded the percentage of transmittance with changing wavenumbers to provide the infrared spectra.

### 2.3. Scanning Electron Microscopy Combined with an Energy-Dispersive X-ray Microanalysis

The surface alterations of the clear aligner samples (5 × 5 mm squares) were observed using scanning electron microscopy (SEM) (Carl Zeiss SMT Ltd., Cambridge, UK). The samples were sputter-coated with gold, and images were taken at different magnifications. A low EHT voltage (5 kV) was used for imaging. A silicon drift energy dispersive X-ray (EDX) detector (UltraDry) (Thermo Fisher Scientific, Madison, WI, USA) was used to assess the elemental composition of the morphological alterations. Elemental microanalysis was conducted with a 10 kV accelerating voltage, 500 X original magnification, a 300 s acquisition time, and 5% dead time. Quantitative analysis of weight percentages (% wt) of the probed elements was conducted using the “Phi-Rho-Z” matrix correction algorithm using NSS version 3.0 software (Thermo Fisher Scientific, Madison, WI, USA) [9,13,16,17].

#### Statistical Analysis

Quantitative data obtained from the evaluation of the clear aligner hardness and EDX microanalysis were tabulated and analyzed using the Statistical Package for the Social Sciences (SPSS) software (version 26.0; IBM Inc., Chicago, IL, USA). All assessments were performed by one examiner and repeated twice (average values were used) to confirm the reliability, which was tested using the paired t-test. One-way analysis of variance (ANOVA) and Tukey post hoc tests were performed to analyze the differences among the clear aligner systems (Invisalign^®^, Eon^®^, SureSmile^®^, and Clarity^®^) in terms of hardness. The chi-square test was used to examine differences in the elemental compositions of the included samples. Results were considered statistically significant at *p* ≤ 0.05.

## 3. Results

### 3.1. Micro-Hardness

Vickers hardness data presented comparable values with no statistically significant differences (*p* > 0.05) among the four included systems, as shown in Table 1.

### 3.2. Fourier Transform Infrared Spectroscopy (FTIR)

**Figure 1:**
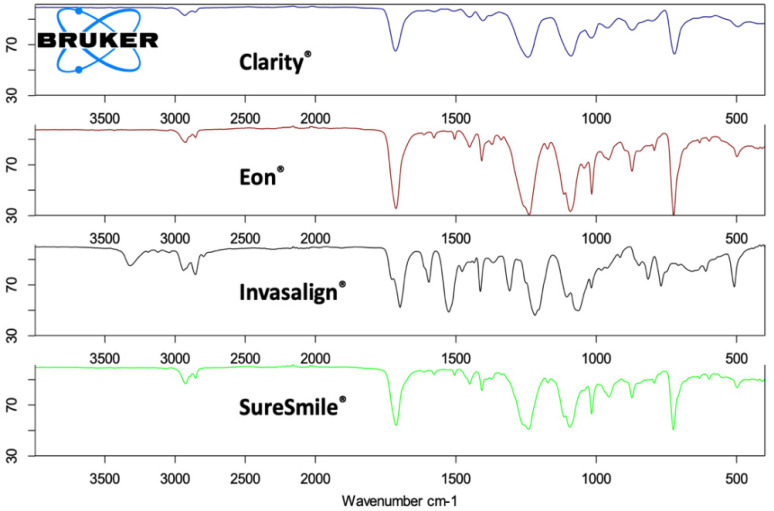
Fourier transform infrared spectroscopy of each clear aligner system.

**Figure 2:**
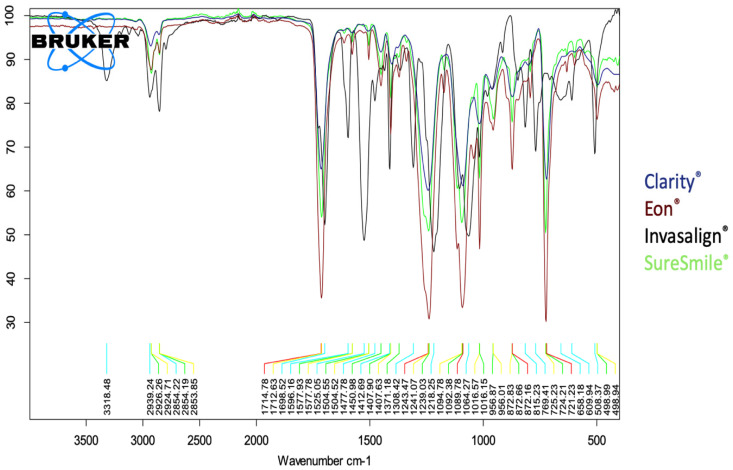
Superimposition of Fourier transform infrared spectroscopy of the four included systems.

Among the four included systems, only the Invisalign^®^ samples presented the characteristic peak of N-H at 3318 cm^−^^1^ and the N-H band at 1525 cm^−^^1^; however, these peaks were not detected in the Eon^®^, SureSmile^®^, or Clarity^®^ samples. The peaks of all four systems were at 2852 cm^−^^1^ and 2939 cm^−^^1^, corresponding to C-H symmetric and asymmetric stretching vibrations, respectively. In addition, a C=O peak observed at 1699 cm^−^^1^, in addition to a peak at 1413 cm^−^^1^, corresponded to the CH_2_ detected in all samples. The peaks appearing at 1218 cm^−^^1^ belonged to the C-O stretching of the ester group in all four samples, which is usually observed in the polyurethane spectrum (Figure 1 and Figure 2).

### 3.3. SEM and EDX

**Figure 3:**
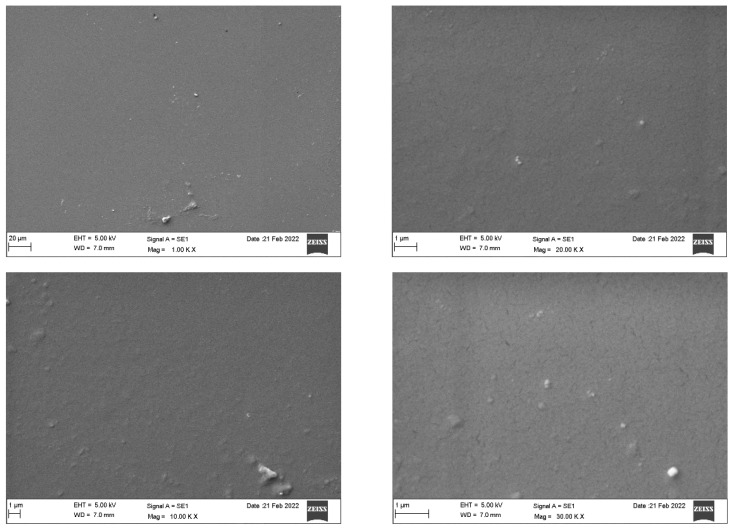
Scanning electron microscopy of the Invisalign^®^ system.

**Figure 4:**
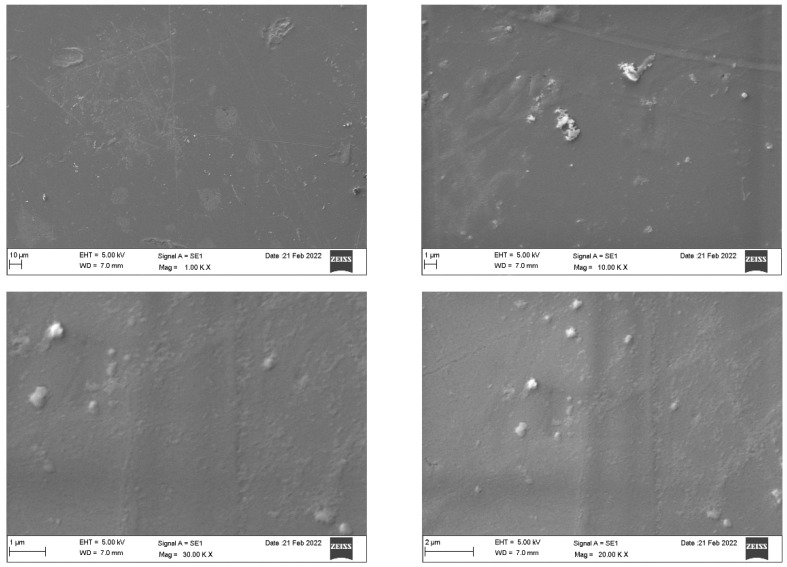
Scanning electron microscopy of the Eon^®^ system.

**Figure 5:**
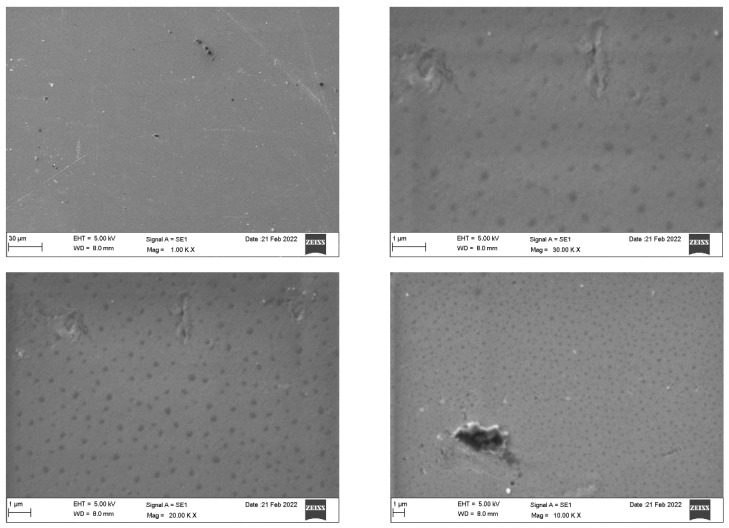
Scanning electron microscopy of the SureSmile^®^ system.

**Figure 6:**
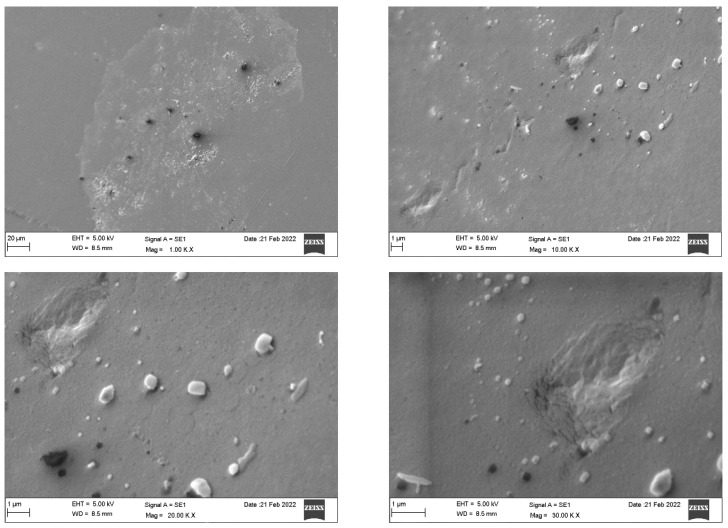
Scanning electron microscopy of the Clarity^®^ system.

SEM analysis of the Invisalign^®^ samples (Figure 3) revealed a homogeneous and dense surface with slightly elevated areas. Impurities were detected as magnification increased. Microcracks were only apparent at higher magnification (30 K). By contrast, the Eon^®^ system (Figure 4) displayed an imperfect rough surface that was filled with irregularities, presenting as grooves, elevations, and depressions of different sizes and shapes with adhered particles. Increased magnification confirmed the irregular and nonhomogeneous structure of the sample. Figure 5 demonstrates the rough, porous surface of the SureSmile^®^ system with multiple grooves and elevations. The porosity of the surface was clearly illustrated at a magnification of 10–30 K. Multiple irregular depressed areas were also observed within the porous background. Lastly, Clarity^®^ scans showed a nonhomogeneous surface configuration with dispersed uneven areas with multiple elevations, depressions, and attached particles. At 10 K magnification, spherical particles and irregularly shaped depressions were readily detected. At higher magnifications (20 and 30 K), extensive roundish defects of different layers indicated imperfect architecture, as shown in Figure 6.

EDX analysis revealed numerous elements, as illustrated in Table 2. Most importantly, carbon and oxygen were present in all four samples. Nitrogen was detected only in Invisalign^®^, whereas mercury was detected in Eon^®^. We found 0.02% and 0.36% fluorine in Eon^®^ and SureSmile^®^, respectively. Minute amounts of sodium and chlorine were found in SureSmile^®^. Statistical analysis showed that the amount of carbon was significantly different among the four systems (*p* = 0.012). However, the percentages of oxygen were similar (*p*-value > 0.05) in all systems (Table 3).

## 4. Discussion

The present study evaluated the structural differences of the Eon^®^, SureSmile^®^, and Clarity^®^ clear aligner systems in comparison to the most commonly used system, Invisalign^®^, using a Vickers indenter, FTIR spectroscopy, and SEM. To the best of our knowledge, the physical and chemical characteristics of Eon^®^, SureSmile^®^, and Clarity^®^ have not been previously reported.

### 4.1. Micro-Hardness

Hardness is the mechanical property of a material’s resistance to a certain load (indentation or penetration). This property can be influenced by several factors, such as the thickness of the material, thermoforming process, polymer structure, and polymerization process. The Vickers indenter was the method of choice for the polymeric material utilized in this experiment [13,18,19]. The Vickers hardness results clearly showed that all tested systems had similar hardness values, as confirmed by the absence of statistically significant differences among the four clear aligner systems (Table 1). In contrast to the similarities in hardness found in our study, an older investigation reported the superior hardness of Invisalign^®^ compared with the other included systems [20].

Invisalign^®^ is the only previously investigated system, and the studies in question included controversial data regarding aging and hardness. Two previous studies concluded an increased aligner hardness after intraoral aging of the retrieved sample [9,21]. On the other hand, *Bradley* et al. reported a lower hardness value after intraoral exposure [22].

### 4.2. FTIR Spectroscopy

FTIR analysis of Invisalign^®^ Eon^®^, SureSmile^®^, and Clarity^®^ revealed comparable spectral profiles on the surfaces of the included samples. The confirmed peaks of the ester group were regularly observed in the polyurethane spectrum for all four systems studied. Our findings are in agreement with recent investigations in which the authors identified a polyurethane-based material of the tested clear aligner samples from the Invisalign^®^ system [20,23,24]. Some mild molecular differences, possibly attributed to the variable molecular composition among the manufacturing companies, were noted. Hence, the characteristic peaks of N-H at 3318 cm^−^^1^ and 1525 cm^−^^1^ were found only in the Invisalign^®^ sample, in accordance with multiple reports by researchers in the literature [20,22,23,24].

### 4.3. SEM and EDX

Invisalign^®^ presented superior structures under the SEM, with a smoother and less defective surface. The other three systems showed a comparable architecture, presenting some degree of impurities, irregularities, and adherent particles on their surfaces. Among the four systems, SureSmile^®^ was the most porous. In 2021, an SEM characterization study of two generations of clear aligners revealed that polymeric materials used in clear aligner fabrication presented chemical stability even after accelerated aging in the laboratory. Most of the reported changes were related to the intraoral usage of the aligners by the patients [17].

The EDX analysis showed that the two main elements (carbon and oxygen) were present in all four systems. These elements constituted the majority of the weight percentages. Statistical analysis illustrated a statistically significant difference in the carbon weight among the four systems, with the highest percentage found in SureSmile^®^ (72.78%) and the lowest percentage found in Invisalign^®^ (39.52%). For oxygen, no statistically significant differences were observed among the systems. Other elements have also been identified in particular systems. For instance, the presence of nitrogen in Invisalign^®^ is considered a characteristic feature that has long been reported in the literature [23,24]. The current experimental FTIR data of the Invisalign^®^ sample coincided with the EDX in reporting nitrogen, which was lacking in the other systems. Negligible percentages of fluorine were found in Eon^®^ and SureSmile^®^, which can be considered beneficial if implemented for the construction of clear aligners in the form of fluoride compounds (sodium fluoride or sodium fluorosilicate). The sodium and chlorine found in SureSmile^®^ were mostly related to the immersion solution of the sample (artificial saliva). Similarly, a previous study reported a chlorine element via EDX analysis, relating its presence to the disinfectant solution used to clean aligners in their experiment [10]. Previous investigations of the Invisalign^®^ product have reported elemental composition changes with decreased carbon amounts and the presence of calcium and phosphorus after intraoral exposure [9,25]. Nevertheless, a considerable amount (17.31%) of mercury was identified only in the Eon^®^ sample, which may contribute to unfavorable biological effects. Tremendous effort has been made since the 1990s to investigate the adverse effects of mercury from dental materials, particularly amalgam fillings. Mercury is a heavy metal known for its toxicity, which depends on the concentration and administration route. Various pathological conditions, including gingivitis, allergic sensitivity, neurological disorders, cardiovascular diseases, and hormonal imbalance have been documented from mercury toxicity [26,27,28]. Further investigation is required to clarify the presence of mercury and its concentration in the clear aligner material. Lastly, the literature currently present on the subject has mainly focused on Invisalign; in this study, other aligners were also evaluated and compared with the former.

## 5. Conclusions

The four included systems, Invisalign^®^, Eon^®^, SureSmile^®^, and Clarity^®^, presented comparable levels of hardness. Some degree of molecular composition differences was presented among the systems; nevertheless, they were all made of a polyurethane-based material. Carbon and oxygen were the main fundamental elements of the studied clear aligners, with nitrogen uniquely present in only the Invisalign^®^ samples. Lastly, when the sample surfaces were evaluated under SEM, Invisalign^®^ had a smoother and more homogenous structure than the other three systems. In general, all included clear aligners had similar characteristics with minimal differences, providing a wide variety of options for clinical orthodontic treatment according to patients’ demands.

## Figures and Tables

**Table 1:** Micro-hardness comparison of the different clear aligner systems via one-way ANOVA and Tukey post hoc tests.

System	Mean ± SD	F-Test	*p*-Value	95% Confidence Interval		Multiple Comparisons (Post Hoc Tukey HSD)			
				Lower Bound	Upper Bound	Invisalign^®^	Eon^®^	SureSmile^®^	Clarity^®^
Invisalign^®^	5.163 ± 0.440	2.449	0.138	4.071	6.255	1	0.143	0.994	0.872
Eon^®^	4.642 ± 0.188			4.176	5.108	0.143	1	0.200	0.382
SureSmile^®^	5.111 ± 0.164			4.703	5.519	0.994	0.200	1	0.955
Clarity^®^	5.003 ± 0.122			4.699	5.307	0.872	0.382	0.955	1

**Table 2:** Elemental composition and weight of each system (EDX microanalysis).

System	Element	Atom Shell (Location)	Element Weight in %
Invisalign^®^	Carbon	K	39.52
	Oxygen		14.13
	Nitrogen		46.36
Eon^®^	Carbon	K	61.54
	Oxygen		21.13
	Fluorine		0.02
	Mercury	M	17.31
SureSmile^®^	Carbon	K	72.78
	Oxygen		24.20
	Fluorine		0.36
	Sodium		1.75
	Chlorine		0.90
Clarity^®^	Carbon	K	69.92
	Oxygen		30.08

**Table 3:** Chi-square analysis of the main elements presented in all four systems.

System	Element	Element Weight in %	Chi-Square	*p*-Value
Invisalign^®^	C	39.52	10.886	0.012 *
Eon^®^		61.54		
SureSmile^®^		72.78		
Clarity^®^		69.92		
Invisalign^®^	O	14.13	5.966	0.113
Eon^®^		21.13		
SureSmile^®^		24.2		
Clarity^®^		30.08		

* Statistically significant, *p* < 0.05.

## Data Availability

The data supporting the reported results analyzed or generated during the study are available upon request from the corresponding author.
